# Advancing the synthesis of trimethylene carbonate: a high-yield green synthesis route

**DOI:** 10.1186/s40643-025-00877-6

**Published:** 2025-04-22

**Authors:** Zisheng Ning, Yuhan Li, Lingmei Dai, Dehua Liu, Wei Du

**Affiliations:** Key Laboratory for Industrial Biocatalysis, Department of Chemical Engineering, Ministry of Education, Tsinghua University, Beijing, 100084 China

**Keywords:** Bio-based chemical, Enzymatic catalysis, Lipase, 1,3- propanediol (PDO), Trimethylene carbonate (TMC)

## Abstract

Trimethylene carbonate (TMC) is an innovative modifier for polylactic acid and a promising biodegradable polymer monomer with broad application potential. However, industrial production of TMC faces challenges such as high catalyst costs, safety issues, and environmental impacts. Enzymatic catalysis offers a potential alternative, but its low product yields have hindered progress. In this study, we introduce a novel synthesis route for TMC using bio-based 1,3-propanediol (1,3-PDO) and dimethyl carbonate (DMC) as substrates. This process involves lipase-catalyzed formation of the intermediate 3-hydroxypropyl methyl carbonate (P1), which is then cyclized to produce TMC. Notably, the by-product, *C*,*C*’-1,3-propanediyl *C*,*C*’-dimethyl ester (P2), reacts with 1,3-PDO to regenerate P1, further enhancing the overall TMC yield. The mechanism exploration reveals that 1,3-PDO acts as both a reactant and an acid catalyst, initiating a nucleophilic substitution reaction on P2 to produce P1. Under optimized conditions, we achieved a total TMC yield of 88%, the highest reported to date.This study provides a novel green synthesis route for TMC that holds great promise for industrial application, given its safer conditions and competitive yields.

## Graphical Abstract

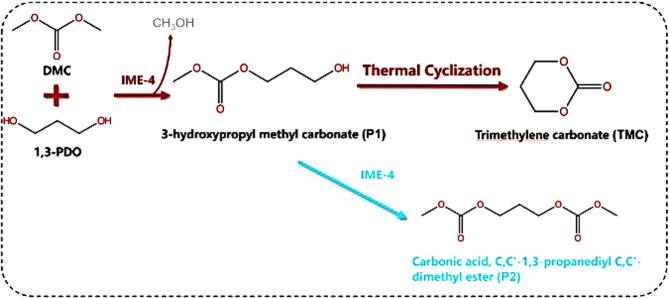

## Introduction

As oil resources continue to diminish and the environmental impact of petroleum-based plastics, often referred to as “white pollution”, escalates, plastic waste has become one of the most pressing global challenges (Moshood et al. 2022a, b). This phenomenon is intensified by the sheer volume of plastic produced annually, with millions of tons finding their way into oceans, rivers, and landscapes, leading to dire ecological consequences. The degradation of these plastics, which take hundreds of years, poses a significant threat to wildlife and human health. In response to this escalating crisis, many countries have enacted policies aimed at limiting the use of specific plastic products. Such measures include bans on single-use plastics and incentives for more sustainable alternatives. This regulatory environment has intensified the urgency for research into environmentally friendly and biodegradable materials that can effectively replace traditional plastics (Flury and Narayan 2021; Filiciotto and Rothenberg 2021). Consequently, the production of biodegradable plastics has surged in recent years. While biodegradable plastics represent only 1% of global plastic production in 2022, their annual growth rate is anticipated to reach 30% by 2025 (Kim et al. 2023; Moshood et al. 2022a, b). This reflects a growing recognition of the need for sustainable materials that align with environmental goals. Among the various biodegradable options, polylactic acid (PLA) has emerged as one of the most commercialized and successful bioplastics. PLA is valued for its excellent biodegradability, biocompatibility, and favorable mechanical properties, making it a leading choice for sustainable materials in a variety of applications, including packaging, disposable tableware, and textiles (Taib et al. 2022). However, despite these advantages, PLA’s low elongation at break and limited toughness restrict its wider application, particularly in areas requiring robust materials that can withstand mechanical stress (Taib et al. 2022; Gao et al. 2011). Research indicates that incorporating trimethylene carbonate (TMC) as a modifier during PLA synthesis can significantly enhance its elongation at break to over 300%. This modification not only improves the flexibility of the material but also allows for adjustments in toughness to suit specific applications, thereby expanding the utility of PLA in diverse fields (Yang et al. 2024; Jiang et al. 2022). Moreover, the PLA synthesized with TMC as a modifier is biodegradable, presenting a more promising developmental pathway compared to other non-biodegradable PLA modifiers that could compromise the environmental benefits of the bioplastic (Xia et al. 2022; Zhang et al. 2017; Hu et al. 2020). 

Furthermore, the unique six-membered ring structure of TMC allows it to undergo ring-opening polymerization, which is a crucial process for producing poly(trimethylene carbonate)-based polymers. These polymers exhibit a slow degradation rate in vivo, a feature that is particularly advantageous in medical applications where sustained performance is necessary. Importantly, TMC-based polymers do not generate strongly acidic by-products during hydrolysis, thus minimizing the risk of inflammatory responses in biological systems. This property is vital, especially in the development of materials intended for use in sensitive environments such as the human body. Additionally, TMC-based copolymers have already found clinical applications, notably as surgical sutures, particularly in pediatric cardiovascular surgery. The ability to tailor the mechanical properties of these copolymers makes them ideal candidates for a range of biodegradable materials that could transform medical applications (Albertson and Sjoling 1992; Zhang et al. 2006; Kricheldorf and Weegen-Schulz 1995; Kéki et al. 2001). The success of TMC in these contexts highlights its potential for broader applications in the medical field, including drug delivery systems and tissue engineering scaffolds (Benicewicz et al. 1991; Yu et al. 2021; Sanson et al. 2010; Bochyńska et al. 2013; Fukushima 2016; Zhang et al. 2011). 

The earliest approach for synthesizing trimethylene carbonate (TMC) involved a gas-phase reaction using phosgene and propylene glycol as substrates. However, this method presents significant safety hazards due to the extreme toxicity of phosgene and the challenges associated with controlling gas-phase reactions (Bowman and Kreutzberger 2003). To address these concerns, some studies have investigated the use of carbon dioxide as a safer carbonyl donor in TMC synthesis, reacting it with oxetane, propylene glycol, or haloalkanes. Among these, the reaction of carbon dioxide with 1,3-PDO produces only water as a by-product, making it environmentally friendly. However, the presence of water significantly inhibits the reaction equilibrium. Honda et al. (Honda et al. 2014) introduced 2-cyanopyridine to react with water, thereby driving the reaction forward. They discovered that cerium dioxide could simultaneously catalyze both reactions, achieving a TMC yield of 79% under conditions of 130 °C and 5 MPa for 12 h. Nevertheless, this reaction requires high pressure, and cerium dioxide tends to aggregate in the solvent during the reaction, reducing its catalytic efficiency. Deng et al. (Deng et al. 2020) used carbon dioxide and bromopropanol as substrates, reacting them in acetonitrile solvent with 3-(Butylimino)-N1,N1,N2,N2-tetracyclohexyl-1-cyclopropene-1,2-diamine as a catalyst at 25 °C and 0.1 MPa for 1 h, achieving a TMC yield of 99%. This method offers mild reaction conditions, but due to the low reactivity of carbon dioxide, the chosen catalyst is highly expensive. Additionally, the separation of CPI-H-Br salt from the reaction products during industrial scaling has not been demonstrated, potentially increasing the complexity of post-treatment steps. Darensbourg et al. (Darensbourg et al. 2010) used carbon dioxide and oxetane as substrates, reacting them in toluene solvent under the combined action of tetrabutylammonium bromide and vanadyl acetylacetonate at 60 °C and 3.5 MPa for 8 h, achieving a TMC yield of 95%. However, the study did not mention the recovery and reuse of the catalyst, which could increase industrial costs. Moreover, the use of toluene solvent poses challenges for subsequent separation and purification, and the reaction’s high-pressure conditions require sophisticated equipment for industrial production. While this approach enhances safety, the low reactivity of carbon dioxide requires the use of highly active catalysts for activation (Honda et al. 2014; Deng et al. 2020; Darensbourg et al. 2010; Zhang et al. 2023; Appaturi et al. 2020; Wu et al. 2024). This necessity leads to stringent reaction conditions or elevated catalyst costs, which impede further development of the process.

Furthermore, Wu et al. (Wu et al. 2024) also synthesized TMC using urea and 1,3-PDO as substrates, catalyzed by zinc chloride and 1-hexadecyl-3-methylimidazolium chloride at 160 °C and 15 kPa for 3 h, achieving a TMC yield of 72%. Although this method offers mild conditions, the use of ionic liquids presents challenges for industrial production. Therefore, there is a continued need for the discovery of a greener, more efficient, and industrially applicable method.

A recent method for synthesizing TMC involves the transesterification of 1,3-propanediol (1,3-PDO) and dimethyl carbonate (DMC), utilizing a diverse array of affordable raw materials. Currently, the predominant industrial catalysts for this reaction are organic lanthanum or organic tin, both of which are expensive and raise safety concerns. Enzymes, particularly lipases, present a promising solution as green and mild biocatalysts capable of facilitating transesterification reactions, positioning them as next-generation catalysts. For example, Hiroaki et al. employed Novozym 435, an immobilized lipase from Novozymes, in acetonitrile as the solvent. Despite adding 600 wt% DMC, the reaction achieved only a 50% yield of TMC after five days (Tasaki et al. 2003). In a novel approach, Pyo et al. employed Novozym 435 in a solvent-free system, achieving a TMC yield of 43.2% through a one-step enzymatic catalysis followed by a thermal cyclization step without a catalyst. While this process is more environmentally friendly and convenient (Pyo et al. 2012), the overall low yield remains a significant barrier to its broader industrial application.

**Scheme 1:**
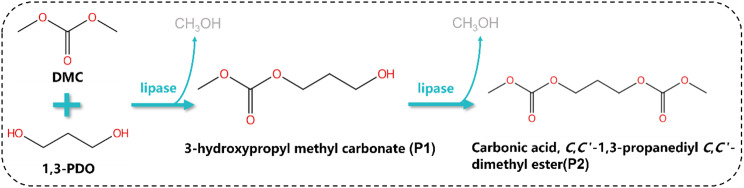
Lipase-catalyzed Reaction Process

To improve the conversion of 1,3-PDO to TMC, it is crucial to minimize the formation of carbonic acid, *C*,*C*’-1,3-propanediyl *C*,*C*’-dimethyl ester (P2) during the enzymatic reaction between 1,3-PDO and DMC to produce the intermediate P1 (Scheme 1). In this study, we present a novel synthesis route for TMC that utilizes bio-based 1,3-PDO and DMC as substrates. This process allows for the conversion of P1 into the final product TMC through cyclization. Furthermore, the by-product P2 reacts with 1,3-PDO to yield P1. Our innovative approach addresses the limitations of traditional enzymatic methods, where the formation of P2 is inevitable and results in low TMC yields. We systematically conducted optimization studies on the enzymatic process, the conversion of P2 to P1, the cyclization of P1 to TMC, and the associated mechanisms.

## Experimental section

### Materials

1,3-propanediol (98%), dimethyl carbonate (98%), molecular sieve (4 Å) and trimethylene carbonate (98%) were supplied by Aladdin. The immobilized lipase IME-4 (enzyme: *Candida antarctica* lipase B, CALB) was from Weifang Kangdi’en Biotechnology Co., Ltd; TL IM (enzyme: *Thermomyces lanuginosus* lipase, TLL), RM IM (enzyme: *Rhizomucor miehei* lipase, RML), Novozym 435 (enzyme: CALB) were all from Novozymes; HEB-1 (enzyme: *Pseudomonas aeruginosa* lipase) was from Mianyang Heben Biotechnology Co., Ltd. All of the chemicals and solvents utilized in this study were obtained from commercial sources without further purification, including ethanol (99.5%), methyl caprate (99%), etc.

### Determination of enzyme activity

The activity of the immobilized lipase was measured by the hydrolysis of tributyrin. The reaction mixtures containing 2 ml of tributyrin and 3 ml of phosphate buffer (PB, 50 mM, pH 7.0) were pre-incubated at 40 ℃ and 200 rpm in a thermostatic shaker for 15 min. Then, a predetermined amount of immobilized lipase was added to start the hydrolysis reaction under the same conditions. After 10 min of reaction, 20 ml of 95% ethanol was added to quench the reaction, and the amount of fatty acid liberated was measured by titration using a 50 mM NaOH aqueous solution. One tributyrin unit (U) was defined as the amount of enzyme that releases 1 µmol of butyric acid per minute under the conditions described.

1 $$\:\begin{array}{*{20}{c}}\begin{gathered} Enzyme\:activity\:{U_I}\left( {\mu \:{\text{mol}} \cdot \:{\text{mi}}{{\text{n}}^{ - 1}} \cdot \:{\text{m}}{{\text{g}}^{ - 1}}} \right) \hfill \\= \frac{{\left( {{V_1} - {V_0}} \right) \times \:{C_{NaOH}} \times \:{{10}^3}}}{{{m_I} \times \:t}} \hfill \\ \end{gathered} \end{array}$$

Where, V_1_ is the titrate value of free or immobilized lipase (mL), V_0_ is the titrate value of the blank (mL), C_NaOH_ is the concentration of NaOH aqueous solution, m_I_ is the mass of immobilized lipase (mg), t (10 min) is the reaction time duration.

### Lipase-catalyzed reaction

Before the reaction, the molecular sieve needs to be activated. To do this, the molecular sieve was placed in a vacuum drying oven at 150 ℃ for 2 h. Once the activation process was complete and the sieve had cooled to room temperature, it was transferred to a desiccator for storage.

In a 100 mL flask equipped with a magnetic stirrer, 2 g 1,3-PDO (approximately 26.28 mmol) was dissolved in the corresponding volume of dimethyl carbonate (with a 1,3-PDO-to-DMC molar ratio ranging from 1:3 to 1:15). Next, 4000 U of immobilized lipase was added to the flask, along with varying amounts of activated molecular sieve (3, 5, 7, or 9 g). The reaction was conducted at 60 ℃ and 200 rpm for 9 h. Samples of 30 µL were periodically extracted for GC analysis to monitor the reaction progress.

### Thermal cyclization reaction

After the lipase-catalyzed reaction, the mixture was filtered to separate the immobilized lipase and molecular sieve. The filtered reaction solution was then subjected to vacuum rotary evaporation at 70 ℃ for 1 h to remove the remaining DMC and methanol. The enzyme-catalyzed product (5 mL) was transferred to a thick-walled pressure-resistant glass tube and stirred during heating. The thermal cyclization reaction was conducted at various temperatures (70–170 °C). A condenser and reflux device were used when the temperature exceeded 130 °C. At regular intervals, 10 µL samples were withdrawn for GC analysis.

### Quantitative analysis

The quantitative analysis of reaction products was performed using gas chromatography (GC, 7890 A, Agilent, USA) equipped with a CP-FFAP CB column and a flame ionization detector (FID). Samples were mixed with 100 µL of a methyl decanoate internal standard solution (10 g/L concentration) and then diluted with anhydrous ethanol to a total volume of 1 mL. The front inlet temperature was set to 245 ℃ with a nitrogen carrier gas pressure of 14.5 atm. The FID detector temperature was maintained at 270 ℃. The initial column oven temperature was held at 110 ℃ for 1 min, then increased to 160 ℃ at a rate of 10 ℃/min and held for 1 min. Finally, the temperature was raised to 260 °C at a rate of 8 °C/min and held for 2 min. The retention times for the respective substances are as follows: 1,3-PDO (3.5 min), P1 (4.5 min), P2 (5.5 min), TMC (8.8 min). We estimated the mass concentrations of the analytes by comparing their peak areas to that of the internal standard (methyl decanoate).

Here, we defined conversion and yield as follows:

2 $$Conversion\, = \,\frac{{Amount\,of\,reactant\,reacted}}{{Amount\,of\,reactant\,input}} \times 100\%$$

3 $${\text{Yield}}\,{\text{ = }}\,\frac{{{\text{Actual}}\,{\text{amount}}\,{\text{of}}\,{\text{product}}}}{{{\text{Theoretical}}\,{\text{amount}}\,{\text{of}}\,{\text{product}}}} \times 100\%$$

### Structural characterization

The structure of the product was characterized by ¹H NMR spectroscopy. The sample (6 mg) was dissolved in deuterated chloroform (CDCl₃) and analyzed on a 600 MHz NMR spectrometer. The observed chemical shifts (δ, ppm) of the target compound were as follows: P1 δ 1.861 (2 H, m), 1.879 (1 H, s), 3.669 (2 H, t), 3.733 (3 H, s), 4.237 (2 H, t); P2 δ 2.058 (2 H, m), 3.792 (3 H, s), 3.797 (3 H, s), 4.253 (4 H, t); TMC δ 2.068 (2 H, m), 4.250 (4 H, t).

## Results and discussion

Using lipase as a catalyst, 1,3-propanediol (1,3-PDO) reacts with dimethyl carbonate (DMC) to produce the intermediate product P1. During this process, P1 often undergoes a transesterification reaction with DMC, also catalyzed by lipase, resulting in the by-product carbonic acid, *C*,*C*’-1,3-propanediyl *C*,*C*’-dimethyl ester (P2). The formation of P2 leads to a low conversion rate of PDO to the final product, TMC.

**Scheme 2:**
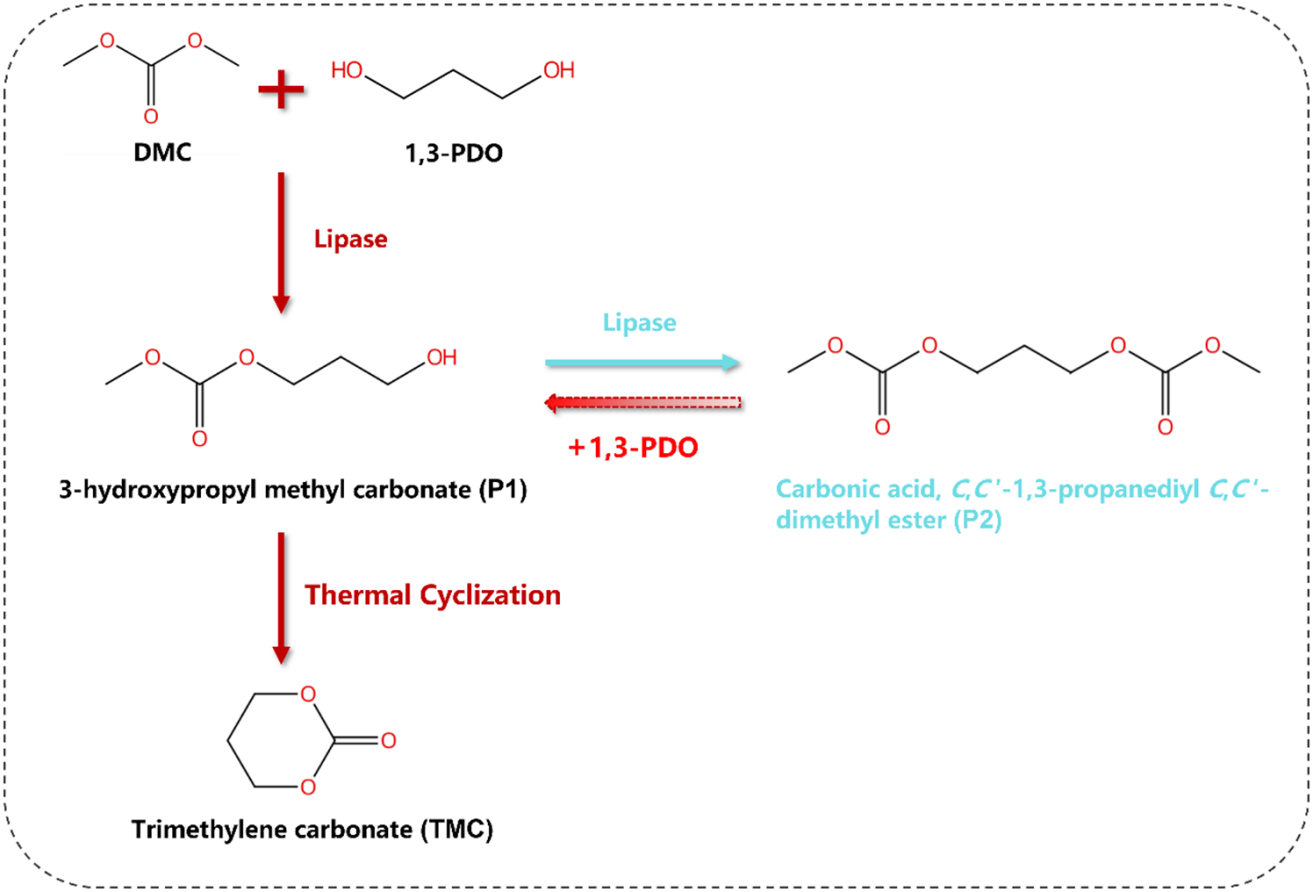
A green route for TMC synthesis

To address the limitations of the traditional enzymatic route, where the formation of by-product P2 reduces the yield of the final product TMC, we proposed a green method for synthesizing TMC, as illustrated in Scheme 2. In this approach, bio-based 1,3-propanediol (1,3-PDO) and dimethyl carbonate (DMC) are used as substrates, with lipase as the catalyst, to produce the intermediate 3-hydroxypropyl methyl carbonate (P1). By adjusting the temperature, by-product P2 can react further with 1,3-PDO to regenerate P1. Subsequently, P1 can be transformed into the final product TMC through cyclization. The following study systematically optimizes the enzymatic process, the conversion of P2 to P1 and the cyclization of P1 to TMC.

### Catalytic performance of different immobilized lipases

**Fig. 1:**
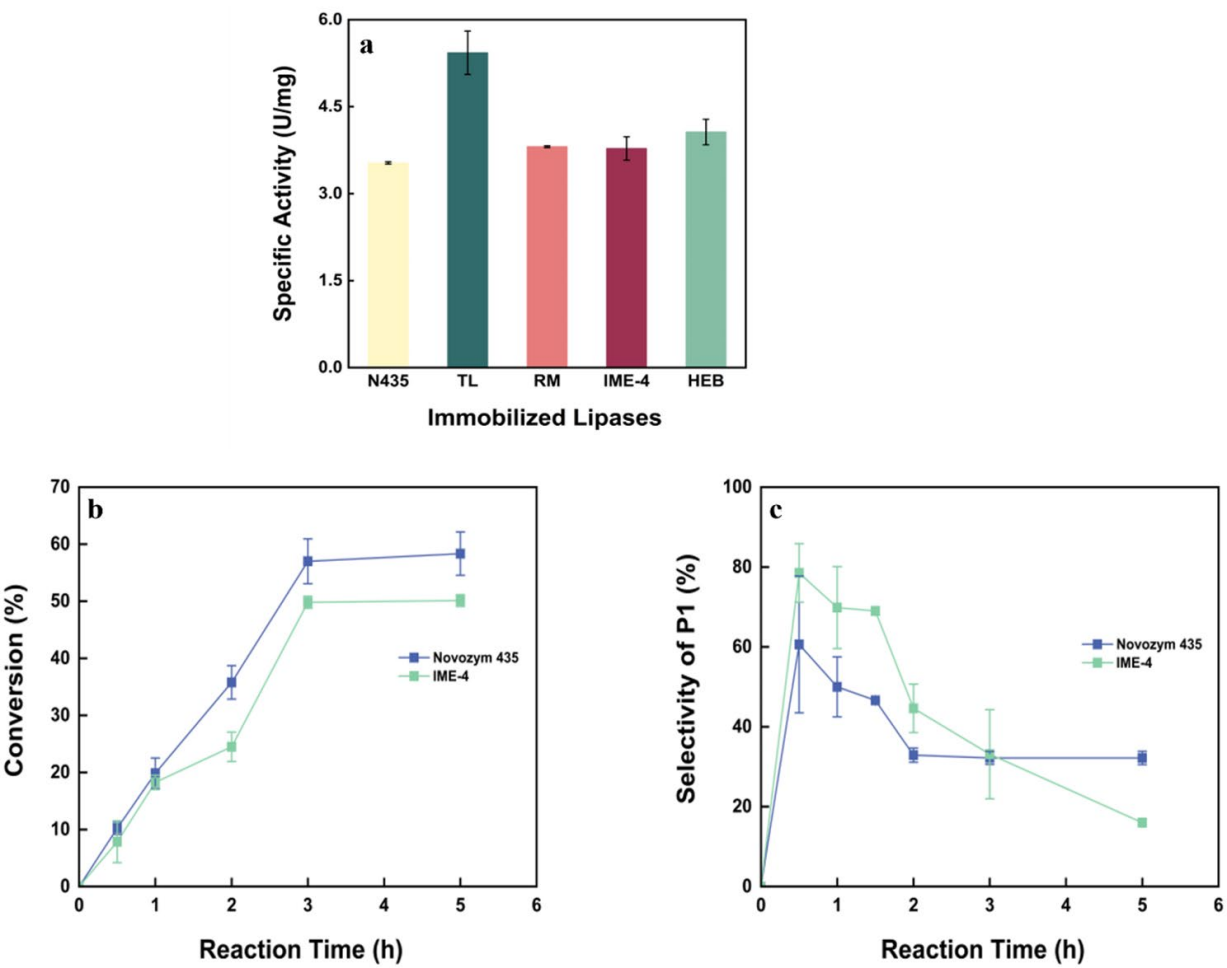
Lipase-mediated reaction between 1,3-PDO and DMC (**a**) Comparison of enzyme activities in the catalytic hydrolysis of tributyrin by different kinds of immobilized lipases. N435: Novozym 435; TL: TL IM; RM: RM IM; HEB: HEB-1. (**b**) The conversion rate of 1,3-PDO over time catalyzed by Novozym 435 and IME-4. (**c**) The selectivity for P1 formation in the catalysis of 1,3-PDO by Novozym 435 and IME-4 over time

In this study, we evaluated five different immobilized lipases to catalyze the conversion of 1,3-PDO and DMC to produce the intermediate product P1. Their catalytic performance was compared, as shown in Fig. 1. Notably, only Novozym 435 and IME-4 effectively catalyzed the reaction between 1,3-PDO and TMC, highlighting the specificity and efficiency of these two lipases. This demonstrates that the lipase CALB immobilized in Novozym435 and IME-4 can effectively catalyze the transesterification between 1,3-PDO and DMC. Under the catalysis of lipase, the transesterification reaction occurs, where PDO and DMC react to form P1, a crucial intermediate product in the production process. Furthermore, P1 can undergo additional reactions with DMC, leading to the formation of a by-product, designated as P2. This conversion to P1 is not only essential but also necessitates a thorough consideration of both the reaction rate and selectivity to ensure optimal yield.

Due to differences in the choice of immobilization carriers, even the same free lipase may exhibit variations in the exposure of active sites, which further explains the differing catalytic performance observed between Novozym 435 and IME-4. Figure 1 indicates that Novozym 435 exhibited a higher reaction rate compared to IME-4, while IME-4 demonstrated greater selectivity for P1 than Novozyme 435. Given that the overarching goal of enzyme-catalyzed reaction is to achieve a high yield of P1, the choice of lipase becomes pivotal. Additionally, when considering the economic aspects of enzyme usage, we found that IME-4 is less expensive than Novozym 435. These factors led us to select IME-4 for further research, as it offers a favourable balance between cost-effectiveness and product selectivity.

### Influence of molecular sieve addition on lipase-catalyzed reaction of 1,3-PDO and DMC

As illustrated in Scheme 1, the enzymatic reaction of 1,3-PDO with DMC produces methanol as a by-product. Research conducted by Pyo and Song (Pyo et al. 2012; Kim et al. 2007) shows that molecular sieves with an average pore diameter of 4 Å or larger effectively absorb the methanol with a diameter of 3.8 Å generated during this reaction, thereby favoring the formation of the desired product. In contrast, the molecular diameters of the reaction substrates, 1,3-PDO and DMC, are both greater than 5 Å, preventing their absorption by the 4 A molecular sieve. In this study, we examined the influence of molecular sieves (4 Å) on the lipase-mediated reaction to produce P1. Before all, to confirm that the molecular sieve does not exhibit catalytic activity, we conducted a blank control experiment in the early stages, where the reaction was carried out without the addition of the enzyme. No reaction was observed under these conditions, further verifying that the reaction is catalyzed exclusively by the immobilized lipase.

**Fig. 2:**
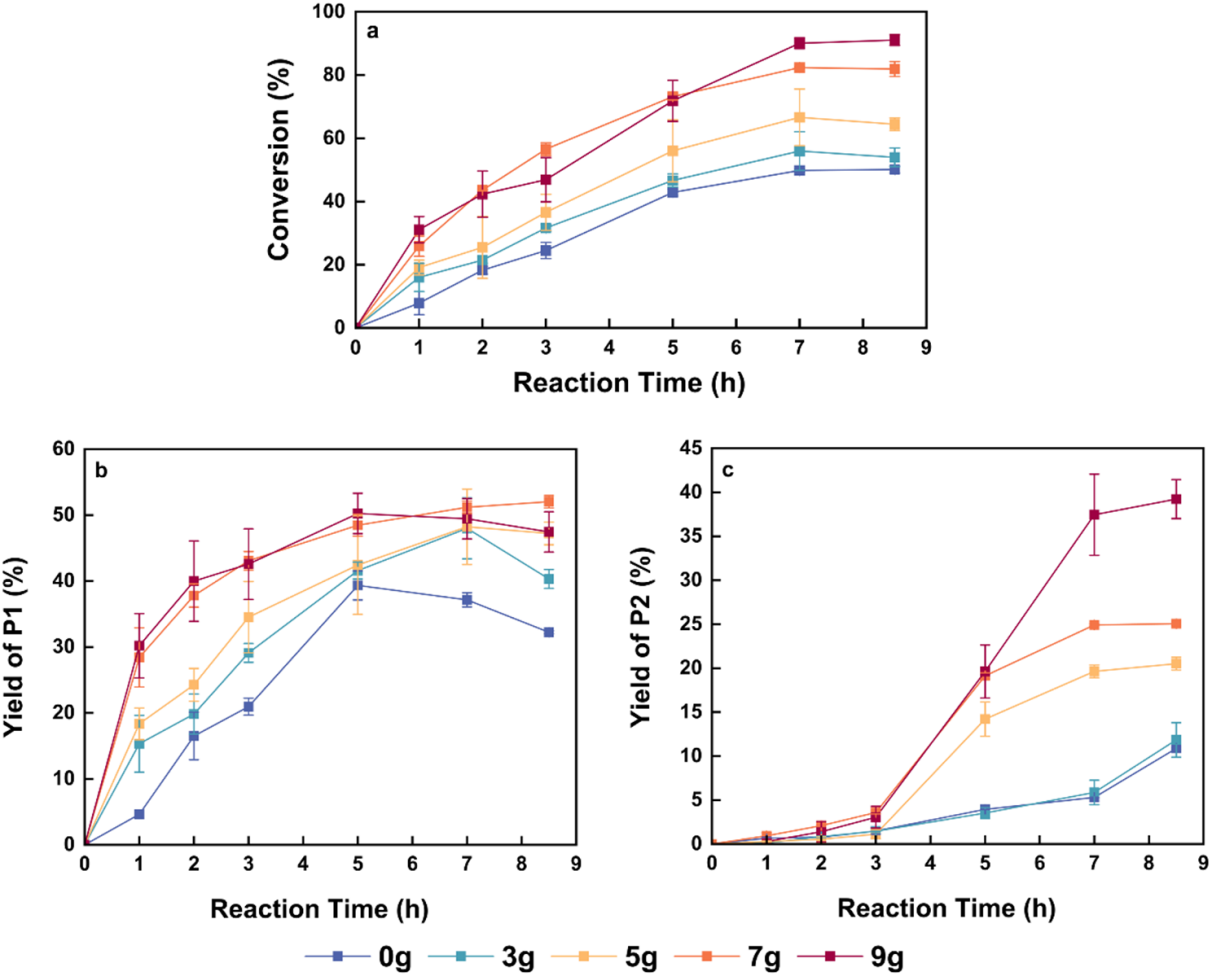
Influence of molecular sieve addition on lipase-mediated reaction of 1,3-PDO and DMC (**a**) Conversion rate of 1,3-PDO under different dosages of molecular sieves, with the dosage of molecular sieves standardized to every 2 g of 1,3-PDO feedstock. (**b**) The yield of intermediate product P1 over time under varying amounts of molecular sieve addition. (**c**) The yield of by-product P2 over time under varying amounts of molecular sieve addition

Figure 2 depicts the influence of varying amounts of molecular sieve on the reaction under the following conditions: 2000 U/g PDO of IME-4, a molar ratio of PDO to DMC of 1:6, 60 °C, and 200 rpm. The molecular sieve amounts were based on the limiting substrate, PDO, with 0, 3, 5, 7, and 9 g of molecular sieves per 2 g of PDO. Equilibrium was reached after 7 h across all conditions. Increasing the amount of molecular sieves enhanced the efficiency of methanol removal, significantly shifting the equilibrium toward product formation and resulting in a higher conversion of 1,3-PDO at equilibrium. When the addition of molecular sieve exceeded 7 g, the equilibrium conversion of PDO surpassed 80%. During the first 5 h of the reaction, the selectivity for P1 and P2 remained stable with 7 g and 9 g of molecular sieves. However, after 5 h, further additions of molecular sieve continued to drive the equilibrium forward. While the first step of the reaction reached equilibrium, the conversion of P1 to P2 had not yet plateaued. As a result, the yield of P2 continued to increase with additional amounts of molecular sieve, while the yield of P1 remained nearly unchanged. Therefore, to achieve a high yield of P1 for subsequent thermal cyclization, the optimal amount of molecular sieve was determined to be 7 g per 2 g of 1,3-PDO.

### Influence of substrate molar ratio on lipase-catalyzed reaction of 1,3-PDO and DMC

**Fig. 3:**
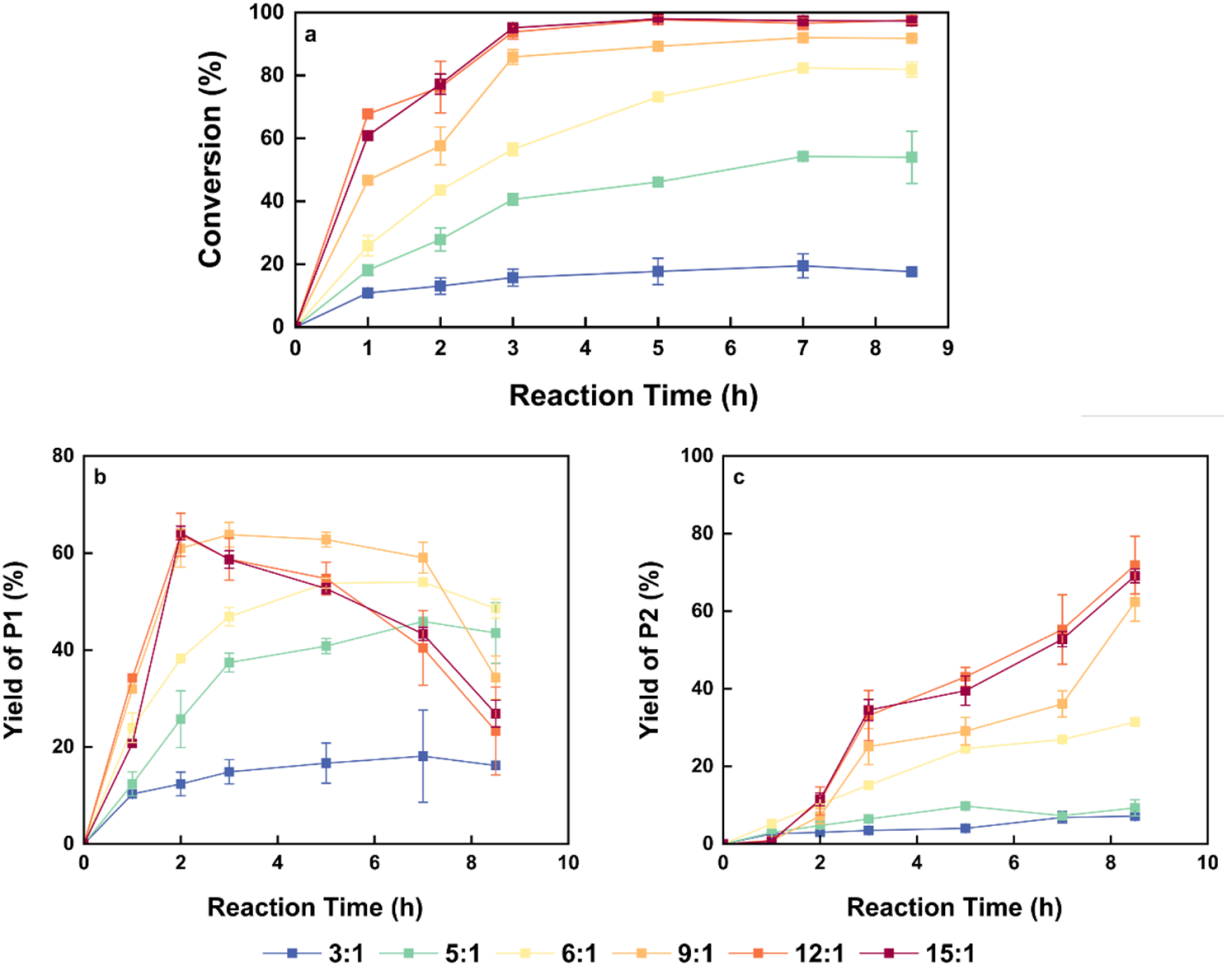
Influence of substrate molar ratio on lipase-mediated reaction between 1,3-PDO and DMC (**a**) The conversion rate of 1,3-PDO over time at different molar ratios (DMC: 1,3-PDO). (**b**) The yield of intermediate product P1 over time at different molar ratios (DMC: 1,3-PDO). (**c**) The yield of by-product P2 over time at different molar ratios (DMC: 1,3-PDO)

In addition to the removal of by-product methanol, the molar ratio of substrates significantly impacts the reaction equilibrium. The high boiling point and viscosity of 1,3-PDO can create considerable contact resistance between enzymes and substrates in a solvent-free system (Kaur et al. 2012). In contrast, DMC, with a boiling point of only 90 ℃ and lower viscosity compared to PDO, facilitates easier to separate (Bernini et al. 2007; Kim and Lee 2017). Therefore, using the optimized molecular sieve amount of 7 g per 2 g of PDO, we investigated the reaction with 1,3-PDO as the limiting substrate and DMC serving both as a solvent and a reactant. The reaction conditions included 2000 U of IME-4 per gram of PDO, 7 g of molecular sieve per 2 g of PDO, and substrate molar ratio (DMC: PDO) of 3:1, 4:1, 5:1, 6:1, 9:1, 12:1 and 15:1 at 60 ℃ and 200 rpm. After 9 h, the conversion of PDO is illustrated in Fig. 3. As the molar ratio of substrates increases, the equilibrium conversion of 1,3-PDO gradually rises. When the molar ratio exceeds 9:1, equilibrium can be achieved within 5 h, with a conversion exceeding 90%.

We also analyzed the yields of the intermediate product P1 and the by-product P2. As shown in Fig. 3, at a substrate molar ratio of 9:1, the PDO conversion after 3 h is only 85%, which is lower than that observed in higher substrate molar ratio groups; however, the yield of the intermediate product reaches 63.8%. This suggests that a 9:1 molar ratio provides better selectivity for generating intermediate products. Therefore, the condition of a 9:1 molar ratio for 3 h was selected for subsequent experiments.

### Optimization on thermal cyclization

**Scheme 3:**
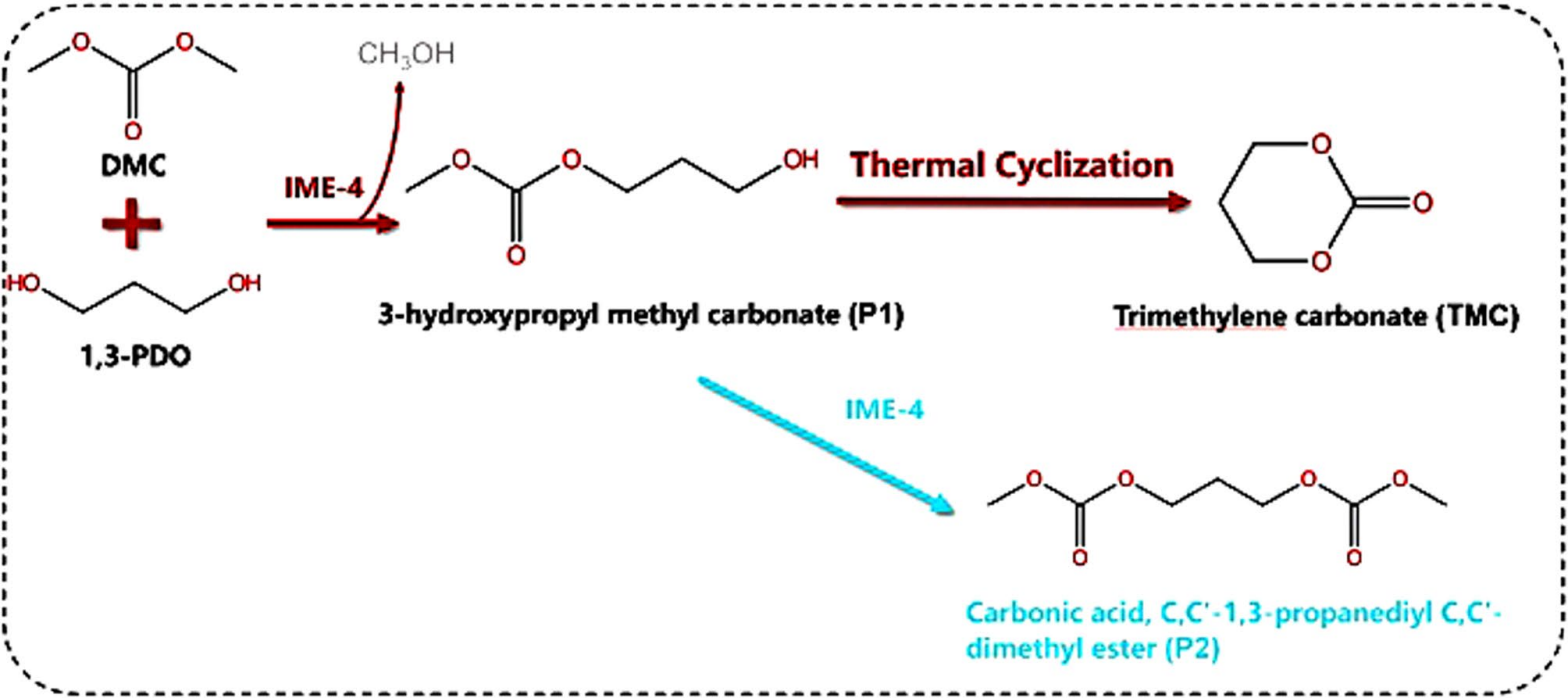
Reaction process from 1,3-PDO and DMC to TMC

During the lipase-mediated production of P1, a by-product, P2, is generated due to enzymatic catalysis. Consequently, it is crucial to promptly halt the enzymatic reaction and convert P1 into the cyclic product TMC through a thermal cyclization reaction (Pyo et al. 2012), as shown in Scheme 3. During this thermal cyclization, methanol remains a by-product that can influence the equilibrium shift towards TMC, while excessive DMC may drive the reaction of P1 towards P2 under heating conditions. Therefore, it is essential to simultaneously remove both methanol and DMC. Since methanol and DMC form an azeotrope, and the azeotropic temperature decreases with lower pressure and a higher proportion of DMC, vacuum rotary evaporation proves effective for the removal of both substances (Hu and Cheng 2017; Liu et al. 2016). 

**Fig. 4:**
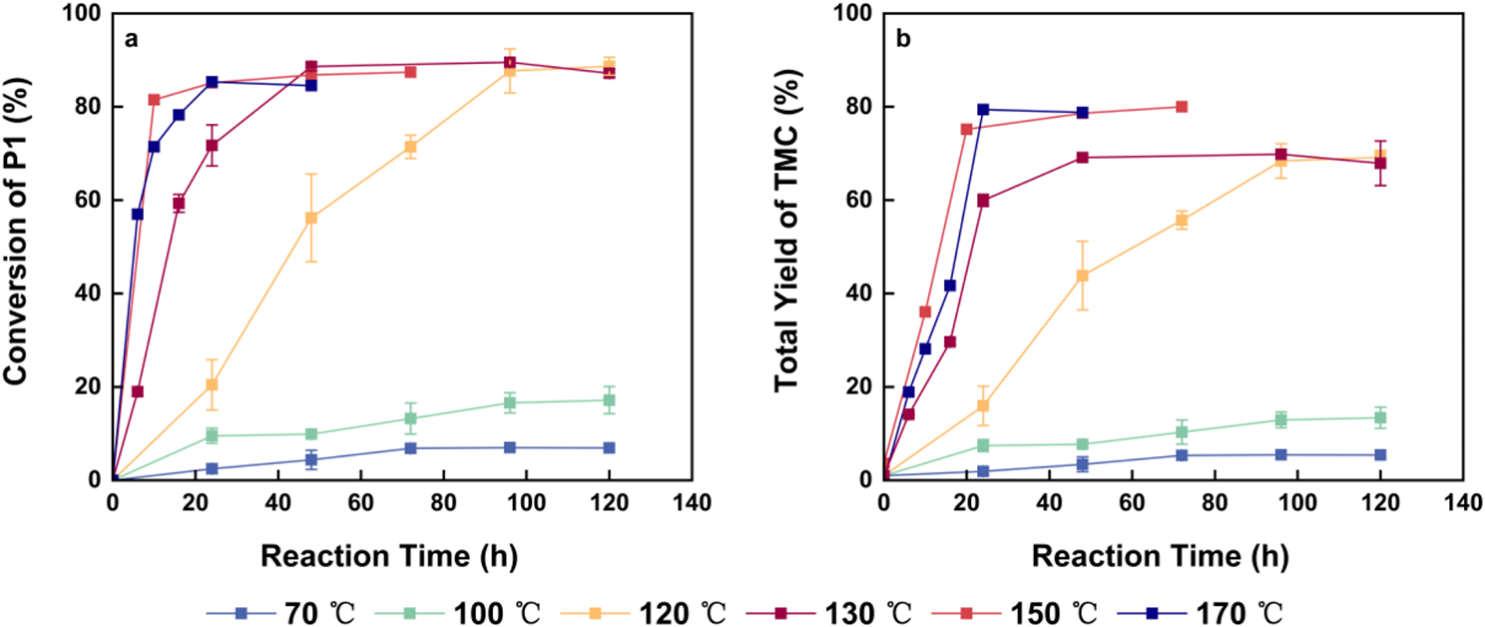
Influence of temperature on the thermal cyclization (**a**) The conversion rate of P1 over time at different temperatures. (**b**) The total yield of TMC from the initial feedstock of 1,3-PDO over time at different temperatures

The thermal cyclization is conducted at temperatures exceeding the boiling point of methanol without the addition of a catalyst, making temperature the most critical factor affecting reaction efficiency. We tested temperatures of 70, 100, 120, 130, 150, and 170 °C. Analysis revealed that when the reaction temperature surpassed 150 °C, GC analysis indicated a decline in the content of P1 and P2. Further GC analysis of the distillate showed that the presence of P1 and P2, suggesting that this temperature exceeds the minimum azeotropic point for these compounds, leading to a reduction of reactants. As a result, a condenser reflux device was incorporated into setups operating above 150 °C. Figure 4 shows the results of thermal cyclization at varying temperatures, demonstrating that higher temperatures effectively promote the conversion of P1, with the reaction rate increasing alongside the temperature. However, improvements beyond 150 °C are minimal. Therefore, 150 °C is identified as the optimal condition for thermal cyclization, achieving an 85% conversion of P1 within 48 h and overall TMC yield of 80%. Notably, the TMC yield relative to P1 was reported as 132%, which is unrealistic, suggesting that other substrates may also be converted into TMC during this process.

**Fig. 5:**
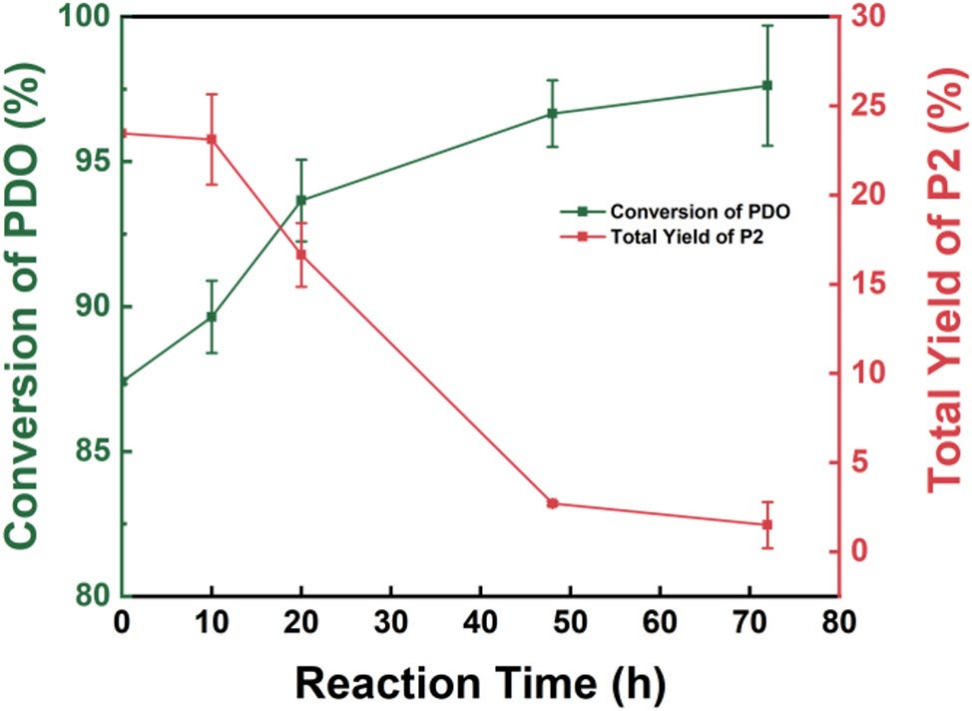
Changes in PDO conversion and total yield of P2 in thermal cyclization (the conversion of 1,3-PDO and the yield of P2 are both calculated based on the total input amount of 1,3-PDO during the first step of the enzymatic catalytic reaction)

Furthermore, calculations of the overall yield of P2 throughout the process reveal a significant decrease in P2 content during thermal cyclization, as shown in Fig. 5. This suggests that P2 likely undergoes ‘pyrolysis’ into P1 under high-temperature conditions, thereby facilitating further thermal cyclization. Additionally, the overall conversion of 1,3-PDO increases, indicating that 1,3-PDO may also participate in the pyrolysis process of P2.

### Pyrolysis of P2 and its application in thermal cyclization

**Fig. 6:**
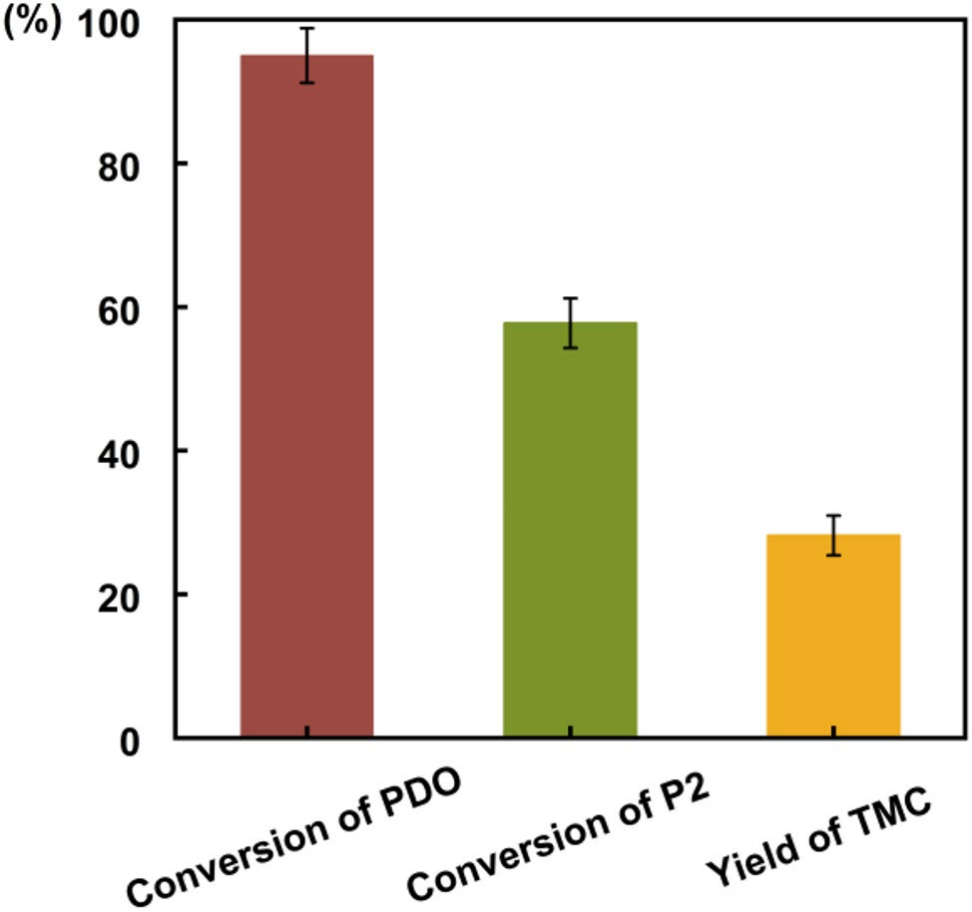
Conversion of 1,3-PDO, P2 and the yield of TMC during the pyrolysis of P2

To further verify the pyrolysis of P2 at elevated temperatures, we referenced the study by Pyo et al., (Pyo et al. 2016) which indicated that a reaction between 1,3-PDO and DMC at a molar ratio of 1:35, combined with 40% molecular sieves relative to the total system mass, can yield up to 99% P2 when conducted at 120 °C for one hour. Scaling up this reaction, we successfully converted all PDO within five hours, achieving a P2 yield of 90%. After removing excess DMC and methanol, we introduced additional 1,3-PDO (equivalent to half the molar amount of P2) and conducted the reaction at 150 °C under reflux. The results, as shown in Fig. 6, demonstrated complete conversion of PDO within 24 h, accompanied by a decrease in P2 content and a conversion rate of approximately 50%. This suggests that the presence of 1,3-PDO at high temperatures can facilitate the transformation of P2 into P1, which subsequently undergoes intramolecular transesterification to synthesize TMC.

**Scheme 4:**
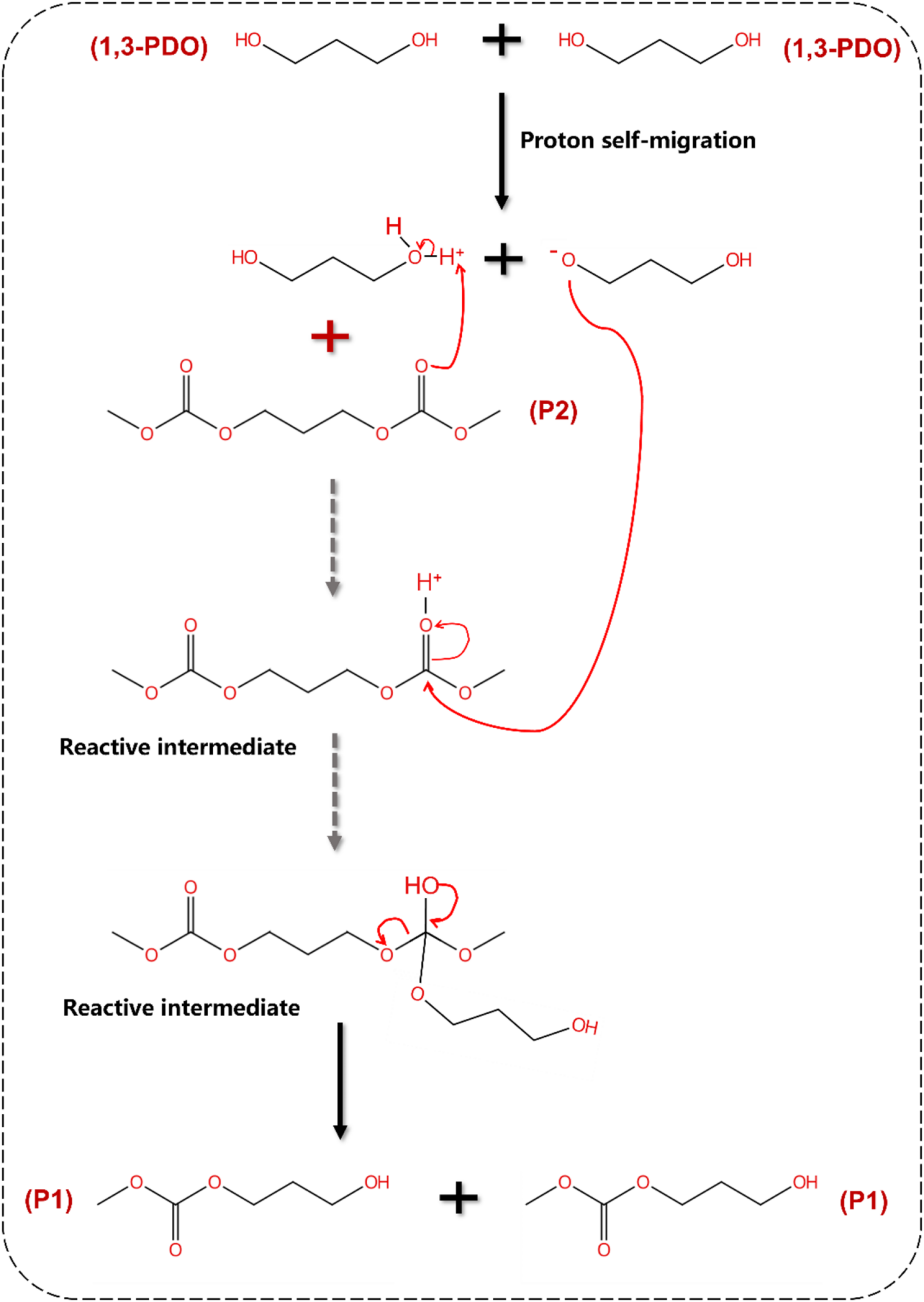
Proposed mechanism for the pyrolysis

This reaction may be attributed to high-temperature conditions that promote a proton self-migration process in 1,3-PDO, releasing protons. In this process, 1,3-PDO functions as both the reactant and an acid catalyst, attacking the carbonyl carbon on P2 and initiating a nucleophilic substitution reaction. As a result, one molecule each of 1,3-PDO and P2 is consumed, yielding two molecules of P1. The proposed reaction mechanism is illustrated in Scheme4 (Guidi et al. 2016). 

**Fig. 7:**
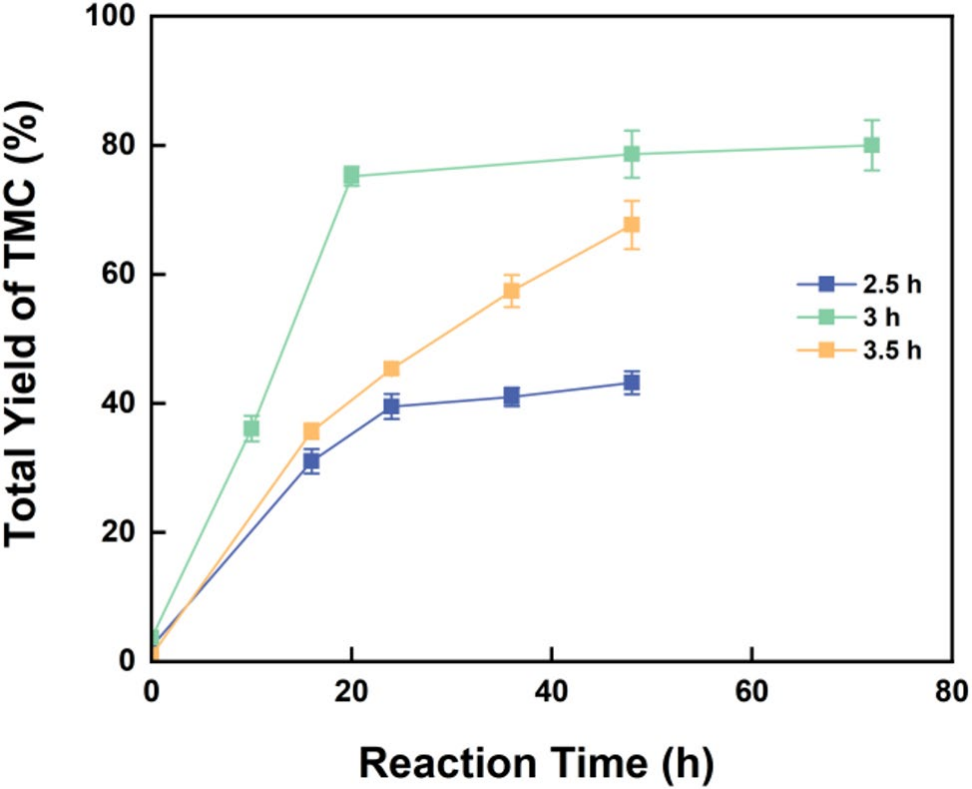
Effect of enzyme catalysis time on TMC yield

Based on the proposed reaction mechanism, it can be inferred that the conversion of PDO during the enzyme-catalyzed process does not need to be excessively high. Retaining some PDO at the initial stage of thermal cyclization can effectively promote the consumption of by-products. Therefore, it is crucial to identify a time point that allows for efficient by-product conversion while ensuring no residual PDO after the reaction. From the earlier optimization results, sampling and thermal cyclization analysis around the 3-hour mark of the enzyme-catalyzed reaction are expected to yield optimal conditions. We selected the reaction results at 2.5 and 3.5 h of enzyme catalysis for thermal cyclization at 150 °C, and the outcomes are shown in Fig. 7. A 3-hour enzyme-catalyzed reaction is the most suitable condition for the thermal cyclization process, as the remaining 1,3-PDO can fully react with P2, further enhancing the overall yield of TMC. Extending or shortening the enzyme-catalyzed reaction time can lead to residual 1,3-PDO or P2, which hinders the improvement of TMC yield. Consequently, considering the pyrolysis of P2, the previously determined enzyme-catalyzed reaction conditions are confirmed to be optimal for the synthesis of TMC via thermal cyclization.

**Fig. 8:**
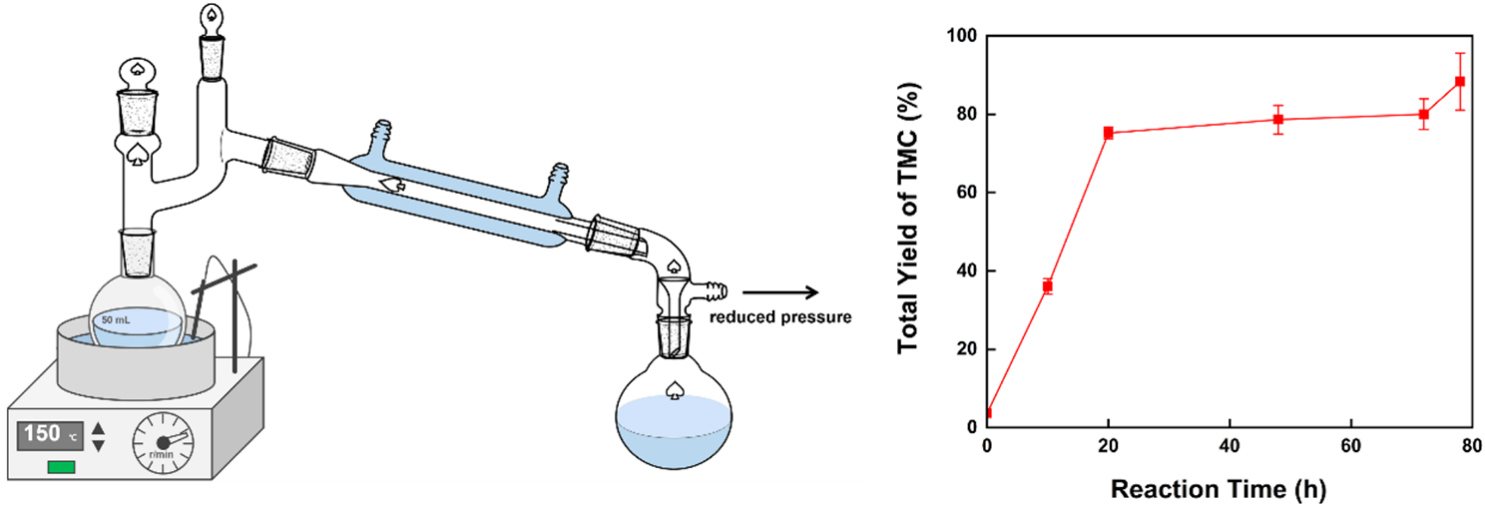
Post-treatment on TMC production and total yield of TMC over time

After optimizing both the enzyme-catalyzed reaction and thermal cyclization, the overall yield of TMC can reach 80%. However, at equilibrium, the conversion of P1 is just 60%, limiting further yield increases. This may be due to the reflux strategy used during heating to avoid azeotrope formation between P1 and P2. Reflux also causes the condensation of by-product methanol, necessitating consideration of post-treatment later in the reaction, when P2 has sufficiently reacted and the boiling point of P1 (199.3 ℃) is well above 150 °C. By removing the reflux setup at this stage, timely separation of methanol becomes possible. As shown in Fig. 8, after post-treatment, the by-product methanol can be efficiently separated, allowing the TMC yield to increase further to 88%.

For product purification, the low boiling point product, methanol, can be removed by vacuum rotary evaporation at 70 °C. Subsequently, recrystallization is performed using a volume ratio of 4:1 of diethyl ether to tetrahydrofuran, resulting in a colorless liquid product with a purity of 95% as determined by gas chromatography.

## Conclusion

This study presents an innovative synthesis route for TMC using bio-based 1,3-propanediol (1,3-PDO) and dimethyl carbonate (DMC) with lipase as the catalyst. The process generates the intermediate 3-hydroxypropyl methyl carbonate (P1), which cyclizes to form TMC. Further, the by-product carbonic acid, *C*,*C*’-1,3-propanediyl *C*,*C*’-dimethyl ester (P2), reacts with 1,3-PDO to regenerate P1, thereby enhancing overall yield. Through the optimization of enzymatic catalysis and cyclization, the total TMC yield reached 88%, the highest reported to date. This method overcomes the limitations of traditional enzymatic routes by reducing P2 accumulation and enhancing feasibility for industrial application. The study simplifies the process through one-step enzymatic catalysis followed by thermal cyclization, providing advantages such as safer, milder conditions and competitive yields.

However, during the enzyme catalysis process, which utilizes a shake-flask system, a large proportion of molecular sieves is used to remove methanol produced during the reaction. Additionally, the reflux in the thermal cyclization process leads to the inhibition of the reaction by the by-product methanol, resulting in a longer reaction time. These two issues are likely to increase economic costs during scale-up. This can be addressed by designing a reactor with a gas outlet at the top, allowing for the direct and continuous removal of methanol during the enzyme catalysis reaction, as the reaction temperature is close to the boiling point of methanol. Similarly, in the thermal cyclization step, reactive distillation can be employed by controlling the distillate head temperature to be higher than the boiling point of methanol, thereby driving the reaction to proceed further. In conclusion, the method employed in this study is more suitable and easier for scale-up in terms of complexity and safety compared to current methods.

## Data Availability

All data generated during this study are included in this article.
